# Medical Society of the State of Pennsylvania

**Published:** 1859-07

**Authors:** 


MEDICAL SOCIETY OF THE STATE OF PENNSYLVANIA.—The eleventh annual meeting of this body was held in the Hall of the University of Pennsylvania, on the eighth of June, Dr. Smith Cunningham, President, in the chair. The delegates, nearly one hundred in number, were welcomed by Dr. Jewell, who made some appropriate remarks on the occasion The annual address was delivered by the retiring president, its subject being the period of Utero-Gestation. Reports were read from the York County and the Philadelphia County Medical Societies, and referred to the Committee of Publication.

On Thursday, Dr. Coates offered resolutions expressive of the regret felt by the Society in the death of Dr. Janney, of this city, and by the medical profession of the State in that of Baron Humboldt. These resolutions were adopted.

Reports of the Beaver, Bradford, and Mercer County Medical Societies were read, and referred to the Committee of Publication

Dr. Worthington, of West Chester, as chairman of a committee appointed on the previous day, read a report, which was adopted without discussion, that it shall be considered as improper for any member of the Society to meet female doctors in consultation.

The following gentlemen were elected officers for the ensuing year:—*President*, Dr. D. Francis Condie, of Philadelphia. *Vice-Presidents*, Dr T. C. Bliss, of Bradford; Dr. John L. Schrack, of Montgomery; Dr. B. F. Schenck, of Lebanon; and Dr. Edw. Wallace, of Berks County. *Corresponding Secretary*, Dr. James Carson, of Philadelphia. *Recording Secretaries*, Drs. John R. Kaul, of Lancaster, and John T. Carpenter, of Schuylkill County. *Treasurer*, Dr. R. P. Thomas. The Censors and Committee of Publication having been appointed, Dr. Cunningham thanked the members for their kindness toward him during his term of office. Drs. Jewell and Worthington then led Dr. Condie, the President elect, to the chair, who made a brief and eloquent address.

The Society adjourned to meet in the City of Philadelphia, on the second Wednesday of June, 1860.

During the session of the Society private evening entertainments were given by several medical gentlemen; and the day after the adjournment of the meeting the delegates took an excursion to Atlantic City, free seats being furnished by the President and Directors of the Camden and Atlantic Railroad Company. An elegant dinner at the Surf House, furnished by the Physicians of Philadelphia, and attended, by special invitation, by the Governor of New Jersey, Dr. Newell, himself a distinguished and zealous practitioner, concluded the festivities of an occasion that will long be remembered for its harmony and social feeling.

